# Calculating Relative Correction Factors for Quantitative Analysis with HILIC-HPLC-ELSD Method: Eight Fructooligosaccharides of *Morinda Officinalis* as a Case Study

**DOI:** 10.1155/2022/8022473

**Published:** 2022-08-12

**Authors:** Lihong Zhou, Hui Ni, Linlin Zhang, Wenyong Wu, Tengqian Zhang, Qi Su, Jing Zhou, Huali Long, Jinjun Hou, Jiyu Gong, Wanying Wu

**Affiliations:** ^1^College of Pharmacy, Changchun University of Chinese Medicine, Changchun 130117, China; ^2^National Engineering Research Center of TCM Standardization Technology, Shanghai Institute of Materia Medica, Chinese Academy of Sciences, Shanghai 201203, China; ^3^School of Chinese Materia Medica, Nanjing University of Chinese Medicine, Nanjing 210029, China; ^4^University of Chinese Academy of Sciences, Beijing 100049, China

## Abstract

**Objective:**

Because the response of evaporating light scattering detector (ELSD) being in a nonlinear mode, there is no consensus on the method of calculating its relative correction factors (RCF), which limits the application of the quantitative analysis for multi-components by a single marker (QAMS) with LC-ELSD.

**Methods:**

Using eight fructooligosaccharides of *Morinda officinalis* as a case study, the nystose (GF3) as a single standard was adopted to develop a QAMS method to simultaneously determine the other seven fructooligosaccharides with HILIC-HPLC-ELSD method. Six calculation methods of RCF were investigated to select the most reasonable method. The relative error of content between the QAMS and the external standard method (ESM) obtained from 30 batches of samples was used as an indicator to evaluate the six methods. Finally, a chemometrics analysis was performed to find the differential components among MO and its three processing products.

**Results:**

It was first reported that only one calculation method was scientific for calculating RCF for the LC-ELSD method. The RCFs of GF3 to the other seven fructooligosaccharides (GF1–GF8) were obtained as 0.86, 0.91, 0.93, 1.05, 1.15, 1.12, and 1.18, respectively. The QAMS of eight fructooligosaccharides of *Morinda officinalis* was validated with good linearity (*R*^2^ > 0.9998) and accepted the accuracy of 95–105% (RSD < 1.81%) based on nystose. Finally, *Morinda officinalis* and its three processed products were distinguished and could be differed based on the content of the eight fructooligosaccharides.

**Conclusion:**

The scientific calculation method of RCF would be of great significance for developing the QAMS method in Pharmacopoeia when performing the LC-ELSD method.

## 1. Introduction

Quantitative analysis of multi-components by a single marker (QAMS) is a method for obtaining accurate content of many components using only a single reference standard combined with a relative correction factor (RCF) and relative retention time (RRT). It is widely used for the simultaneous determination of multiple components in Chinese medicinal materials and preparations, improving and enhancing the quality standards of Chinese medicines and ensuring safe and effective clinical use, while greatly cutting the cost of additional analytes for quantitative methods. RCF is the ratio of the correction factor (*f*) of the reference to the component to be measured. The *f*-value reflects the proportionality between the quantity (mass or concentration) of the constituent and the response value of the detector over a specific linear range. Detectors typically used for quantitative analysis are ultraviolet absorption detectors (UV) and evaporative light scattering detectors (ELSD). In the UV detector, the f-value is the slope of the standard curve, as in the case of saponins, isoflavonoids, and glycosides in red *ginseng* and *Astragali* Radix [1, 2]. Since the response of the ELSD is in a nonlinear mode, there is no consensus on the calculation method of its RCF [3]. It is necessary to select the optimal RCF calculation method and establish the accurate QAMS method for the ELSD system, which is of great significance for the improvement and formulation of pharmacopoeia.

*Morinda officinalis* Radix (MO) is the dried root of *Morinda officinalis* How, sweet and pungent in taste, slightly warm. It has the efficacy of tonifying the liver and kidney, strengthening the muscles and bones, and dispelling wind and dampness [4]. In the Chinese Pharmacopoeia (ChP.2020), MO was processed into three kinds of product, steam-processed MO (StMO, St), salt-steamed MO (SMO, S), and licorice-boiled MO (LMO, L), for enhancing its effects. MO contains various chemical components such as polysaccharides, oligosaccharides, anthraquinones, iridoid glycosides, and organic acids [5–7]. Studies have shown that fructooligosaccharides (GFns), with anti-depressant, anti-aging, anti-osteoporosis, and other activities, are one of the main types of active ingredients in MO [8–12]. In the current standards, such as ChP.2020 and Hong Kong Chinese Materia Medica Standard (Volume X), the content of nystose (GF3) is used as an indicator for the quality control of raw and its three processed products, with a lower limit of 2.0% and 2.3%, respectively [13]. It is worth noting that in addition to GF3, the MO also contains many other fructooligosaccharides, such as sucrose (GF1), 1-Kestose (GF2), 1F-fructofuranosylnystose (GF4), 1,1,1,1-kestohexose (GF5), fructoheptasaccharide (GF6), fructo-oligosaccharide DP8/GF7 (GF7), and fructo-oligosaccharide DP9/GF8 (GF8) with high content and physiological activity [14, 15]. Therefore, for the improvement of the quality standard of MO and its processed products, the quality should be comprehensively evaluated by the content of GFns rather than that of GF3.

Quality control studies on the GFns components of MO have been performed in the literature and the qualitative/quantitative detection methods were established on UHPLC-ELSD [16], HPLC-CAD [17], NIR [18], and LC-MS [19], among which UHPLC-ELSD was widely used. In addition, the hydrophilic interaction chromatography (HILIC) system is a primary method for separating GFns [20]. For example, Y. Yu determined GF1 to GF11 in MO, StMO, SMO, and L. Yang determined GF1 to GF4 in different parts of MO, in both of which HILIC-HPLC-ELSD was used [20, 21]. However, the content of GFns in MO was all calculated by the external standard methods in the existing literatures, in which many expensive standard substances were required. Therefore, it is urgent to establish a QAMS method based on the HILIC-HPLC-ELSD system to evaluate the GFns of MO and its processed products.

Based on the previous studies, the calculation method of RCF in the HILIC-HPLC-ELSD system was investigated with MO as an example. The QAMS methods with six RCF calculation methods for the eight oligosaccharides in ELSD were established and compared with the external standard method to select the optimal RCF. Subsequently, the eight fructooligosaccharides from 30 samples were analyzed by chemometrics to reveal their variations during steaming- and boiling-process.

## 2. Materials and Methods

### 2.1. Materials

Eight standard substances were employed: Sucrose (GF1, purity: 99.8%) was purchased from China National Institutes for Food and Drug Control (Shanghai, China); 1-Kestose (GF2, purity: 98%) and 1F-fructofuranosylnystose (GF4, purity: 80%) were ordered from Wako Pure Chemical Company (Sichuan, China); Nystose (GF3, purity: 90.8%) was purchased from Shanghai Standards Biotech (Shanghai, China); 1,1,1,1-Kestohexose (GF5, purity: 98.0%) was purchased from Sichuan Weikeqi Bio-Technology (Sichuan, China); Fructoheptasaccharide (GF6, purity: 99.52%), Fructo-oligosaccharide DP8/GF7 (GF7, purity: 99.52%), and Fructo-oligosaccharide DP9/GF8 (GF8, purity: 99.21%) were purchased from ChenDu MUST Bio-Technology (Sichuan, China). Acetonitrile (Merck, Germany) was chromatographically pure and ultrapure water was prepared by Milli-Q ultrapure water system.

Three batches of MO samples were purchased from different regions of China. The 27 batches of products processed with steam, licorice, and salt were prepared from the three batches of MO with triplicate parallel samples for each product by Gansu Tianshili Zhongtian Pharmaceutical Co., Ltd. According to the ChP. 2020. The information was shown in Table 1 and Figure S1.

### 2.2. Preparation of Standard Solutions

GF2 was accurately weighed and dissolved in 60% ethanol (v/v) to obtain a standard stock solution with a concentration of 1043.46 *μ*g/mL. Reference standards (GF3 to GF8) were weighed accurately and transferred to a 5 mL volumetric bottle. Then precisely draw 3.5 mL of GF2 stock solution into the 5 mL volumetric flask and diluted by 60% ethanol (v/v) to the volume to obtain a mixed standard solution with a concentration of 730.422 *μ*g/mL (GF2), 1180.400 *μ*g/mL (GF3), 950.40 *μ*g/mL (GF4), 1130.920 *μ*g/mL (GF5), 1212.154 *μ*g/mL (GF6), 1162.394 *μ*g/mL (GF7), and 1035.752 *μ*g/mL (GF8), respectively. The mixed stock solution was diluted to 1, 1.5, 2.2, 4, and 10 times to make a series of standard solutions. In addition, a series of GF1 standard solutions with concentrations of 934.128 (cal-5), 653.890 (cal-4), 420.358 (cal-3), 256.885 (cal-2), and 116.766 (cal-1) *μ*g/mL were obtained.

### 2.3. Preparation of Sample Solutions

All samples were powdered using a pulverizer and passed through a 24-mesh sieve. The powdered sample (0.25 g) was dissolved in 25 mL of 60% ethanol (v/v) in a conical flask with a stopper, weighed, and sonicated (250 W, 53 kHz) for 10 minutes, and allowed to cool. The mixture was weighed again and replenished the lost weight with the same solvent. The supernatant was passed through a 0.45 *μ*m nylon66 membrane and the successive filtrate was collected as the sample solution for further analyses.

### 2.4. HILIC-HPLC-ELSD Analysis

The quantitative assay was performed on an Agilent 1260 series HPLC system equipped with an ELSD (Agilent Technologies, Palo Alto CA, USA), which was controlled by Agilent ChemStation software (B.04.03-SP1). The separation of analytes was conducted on a Waters XBridge HILIC column (4.6 × 150 mm, 3.5 *μ*m) with a flow rate of 1.0 mL/min at 30°C. The mobile phases were acetonitrile (A) and water (B) with a gradient elution of 88% A at 0–1 min, 88–78% A at 1–10 min, 78–65% A at 10–20 min, 65–88% A at 20–20.1 min, and 88% A at 20.1–35 min. The injection volume was 5 *μ*L. The drift tube temperature of ELSD was 50°C and the nitrogen cumulative flow rate was 1.0 mL/min.

### 2.5. Calculation of Relative Correction Factors

As shown in Table 2, six methods were employed to calculate the RCFs in ELSD. F_k_ was the slope of the linear equation of other seven analytes, C_k_ was the true concentration of other seven analytes in standard solution, C_k−detected_ was the concentration of other seven analytes calculated by calibration curves of nystose (GF3) in standard solution, and A_k_ was the peak area of other seven analytes in standard solution. F_s_ was the slope of the linear equation of nystose (GF3), C_s_ was the concentration of GF3 in standard solution, and A_s_ was the peak area of GF3 in standard solution.

### 2.6. Chemometrics Analysis

The Hierarchical cluster analysis (HCA) was carried out by calculating Squared Euclidean distance with Origin software (2021). Principal component analysis (PCA) and orthogonal partial least-squares discrimination analysis (OPLS-DA) were performed with SIMCA software v.14.1 Umetrics, Umea, Sweden, and components with VIP values >1.0 in OPLS-DA were defined as potential chemical markers and applied for further analysis.

## 3. Results and Discussion

### 3.1. Optimization of Sample Preparation

Taking the theoretical plates and tailing factors of GF3 and the extraction efficiency of the eight components as indicators, sample preparation was optimized systematically concerning extraction solvents (ethanol (20%, 40%, 60%, and 80%, v/v), methanol (20%, 40%, 60%, and 80%, v/v), and water), extraction methods, extraction solvent volumes, extraction time, and extraction frequency. The ultimate choice was 60% ethanol (v/v) as the extraction solvent, 25 mL as the extraction solvent volume, and 10 min as the ultrasonic extraction time.

### 3.2. Optimization of Chromatographic Conditions

As the GFns component was strongly hydrophilic and had no UV absorption, the HILIC-HPLC-ELSD system was performed in this research, among which HPLC-ELSD has been used to determine GF3 in both ChP.2020 and Hong Kong Chinese Materia Medica standard (Volume X).

The separation of GFns has investigated in three columns, XBridge HILIC (silyl group), ACHROM XAmide (amide group), and ZIC HILIC column (amphoteric group). The XBridge HILIC column was selected ultimately for its smoother baseline and the shortest retention time of eight GFns in approximately 20 minutes (Figure S2). For the improvement of the efficiency, the column particle size (2.7, 3.0, 5.0 *μ*m), the column temperature (25, 30, 35, 40°C), the flow rate (0.9, 1.0, 1.1 mL/min), and the ratio of acetonitrile in the mobile phase (±1%) were investigated this research. In addition, four ELSD parameters, including the gain value, evaporator temperature, nebulizer temperature, and gas flow rate, were also optimized.

The ultimate chromatographic parameters were as follows: Waters XBridge HILIC column (4.6 × 150 mm, 5.0 *μ*m), mobile phase: acetonitrile and water, flow rate: 1.0 mL/min, injection volume: 5 *μ*L, column temperature: (30 ± 1)°C, ELSD detector: gas: N2, gain value: 1.0, evaporator temperature: 40°C, nebulizer temperature: 50°C, and gas flow rate: 1.6 SLM.

### 3.3. Calibration Curves, Limits of Detection and Limit of Quantification

**Figure 1:**
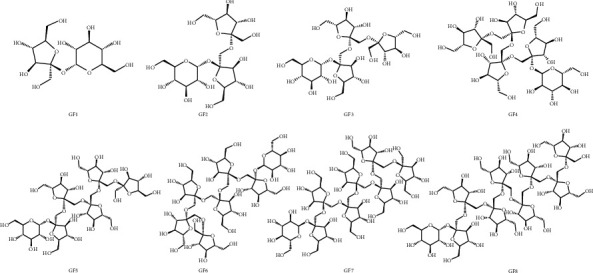
The structures of eight GFns.

**Figure 2:**
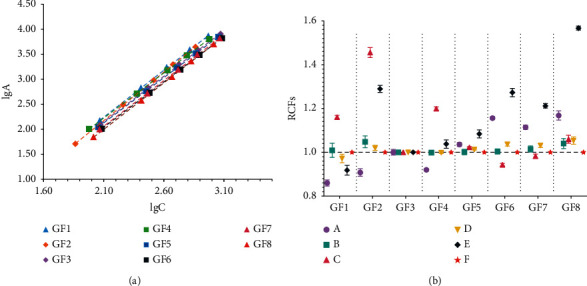
(a) The calibration curves of eight reference standards. (b) The RCFs of eight analytes against GF3 were calculated by six methods.

The structures of eight GFns were shown in Figure 1. The calibration curve, regression coefficients, linear range, limits of detection (LOD), and limits of quantification (LOQ) were shown in Table 3 and Figure 2(a). All calibration curves showed good linearity (*R*^2^ > 0.9998) within the test ranges. In addition, the LOD and LOQ of each standard were in the ranges of 1.29–3.41 *μ*g/mL and 4.32–11.37 *μ*g/mL, respectively, which were 10 times better than the reported results [14], indicating the high sensitivity of the HILIC-HPLC-ELSD system in this study.

### 3.4. Calculation of RCFs with Six Methods

As summarized in Section 2.5, there were six main calculation methods of the RCFs in the HPLC-ELSD system. For establishing a more accurate QAMS method, the RCFs of the analytes were calculated by the six methods. As the quantitative marker of MO in ChP.2020, GF3 had the advantages of moderate retention time, stability, and inexpensive, which was selected as the single marker. The results showed there were significant differences in the RCFs among different methods (Table 4 and Figure 2(b)). Then, further analysis would be carried out to compare them.

### 3.5. Precision, Repeatability, Stability Testing, And Recovery Test

The content determination results of the QAMS methods with six RCFs calculation methods should be compared with those of the external standard method to select the optimal RCFs calculation method. Methodological validation of the method was required prior to the content determination. To evaluate the precision of the instrument, six successive injections of the same sample solution on one instrument were performed. The results (Table 5) showed the relative standard deviation (RSD) of the peak areas was less than 1.91%. The repeatability was tested with nine test solutions covering three different concentration levels (0.125 g, 0.250 g, and 0.375 g).

The RSDs of the peak areas for each analyte were less than 3.2%. The stability was analyzed by storing the sample and standard solutions at room temperature for 0, 6, 12, 24, and 36 hours. The RSDs of the sample and standard solution were less than 2.42% and 2.23%, respectively, indicating both sample and standard solutions were stable over 36 hours.

The recovery experiments were performed by adding three different volumes (5 mL, 10 mL, and 15 mL) of GF3 solution (500 *μ*g/mL) to the flasks with the powder of MO samples (125 mg), and triplicate experiments were performed at each level. The recovery was calculated according to the formula:

(1) $$\begin{array}{c} \text{recovery}=\frac{\text{measured amount}-\text{origin content}}{\text{spiked amount}}\times 100\%. \end{array}$$

As shown in Table 6, the recoveries of spiked GF3 ranged from 95 to 105% (RSD < 1.81%), which indicated the good accuracy of the method.

### 3.6. Selection of the Optimal RCFs by the Comparison of QAMS and External Standard Method

**Figure 3:**
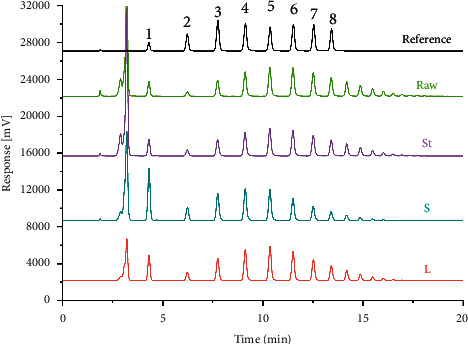
The HPLC chromatograms of eight fructooligosaccharides for MO (Raw) and its three processed products. 1-GF1, 2-GF2, 3-GF3, 4-GF4, 5-GF5, 6-GF6, 7-GF7, and 8-GF8.

As previously mentioned, the optimal RCFs were selected by comparing the content determination results of the QAMS methods with six RCFs calculation methods and those of the external standard method. So, the QAMS and the external standard method were used to determine the content of eight GFns in 30 batches of MO (raw) and its three processed products. The HPLC chromatograms were shown in Figure 3.

**Figure 4:**
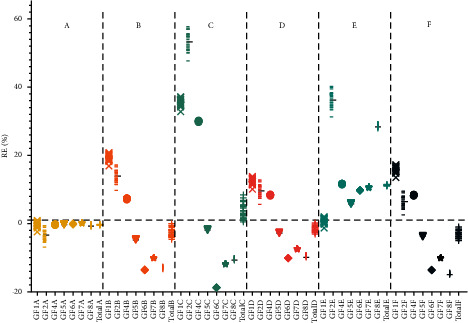
The relative error between the content calculated by the QAMS and the external standard method A, B, C, D, E and F were six RCF calculation methods, respectively.

Then the relative error (RE) between the QAMS and the external standard method was used as the index to evaluate the accuracy of the six QAMS methods. The results (Figure 4) demonstrated that among the six methods, the RE of Method A was closest to zero, illustrating that the established Method A was the most accurate and reliable. Finally, Method A was selected to calculating RCFs in HPLC-ELSD, and the RCFs of GF1-GF8 were 0.86, 0.91, 1.00, 0.93, 1.05, 1.15, 1.12, and 1.18, respectively.

(2) $$\begin{array}{c} \text{RE}\left(\%\right)=\frac{\text{QAMS}-\text{ESM}}{\text{ESM}}\times 100\%. \end{array}$$

### 3.7. The Evaluation of System Suitability on RCFs

The system suitability test of RCFs was investigated on two Agilent 1260 ELSD instruments from different vendors, and the results showed that the RCFs of eight GFns exhibited good repeatability (RSD < 1.8%) (Table 7).

### 3.8. Chemometric Analysis on the Content of the Samples

Subsequently, the content of 30 batches of raw and processed MO samples calculated by the optimal RCFs was used for chemometric analysis.

In the results of the content (Figure S3), it was obviously found that the total oligosaccharides content of one of the three parallel samples L_02-3 (36.22%) was significantly lower than that of the other two parallel samples L_02-1 and L_02-2 (49.86% and 47.33%) which probably came from the preparation process. To find the variation of the oligosaccharides between the raw and processed products, the sample L_02-3 was eliminated in the further analysis, and so was the sample S_02-3.

**Figure 5:**
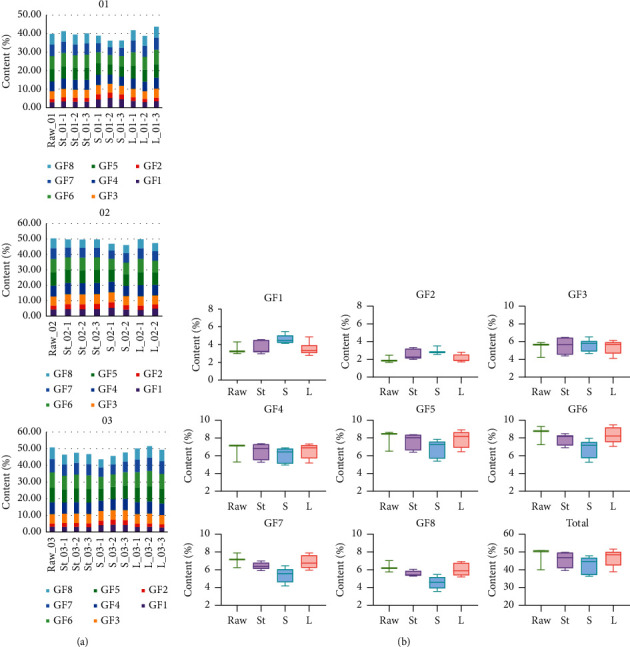
The content of GFns in 28 batches of MO (raw) and its three processed products. (a) Distribution of GFns in three batches of raw (01, 02, and 03) and their corresponding processed products, (b) distribution of each GFn in raw and processed products.

Twenty-eight batches of samples were eventually used for further analysis, including three batches of raw and 25 batches of processed products.  Figure 5 showed the distribution of the content of GFns in 28 batches of samples. As shown in Figure 5(a) and Figure 5(b), the content of the individual and the total oligosaccharides differed slightly between the raw and processed products. It was also found that the contents of GF3 in the four decoction pieces were about 5% (Table S1 and Figure 5), while the total content of the eight oligosaccharides were about 45%, which indicated that it was unreasonable to use GF3 only to evaluate the quality of raw and processed MO.

**Figure 6:**
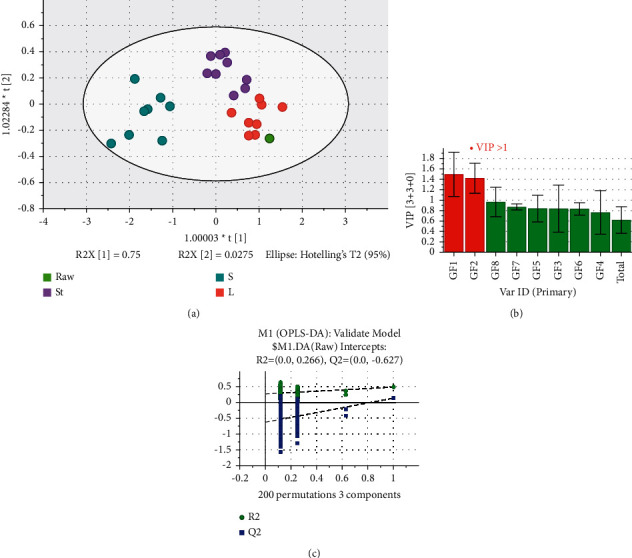
(a) The score plot of OPLS-DA, (b) the VIP plot, and (c) the permutation test for 28 batches of MO and its three processed products.

Since there were differences among the three batches of the raw, the content ratio of the processed products to the corresponding raw samples was used for analysis. HCA could group samples with the same characteristics and determine the variation degree of samples with the same characteristics in the group, which could reveal the differences between the raw and processed products. The HCA analysis, an unsupervised pattern recognition method based on Euclidean distance, differentiated the samples into two major groups in which S was in Group One and St, L, and the raw MO were in Group Two. PCA was utilized to investigate the chemical differences between raw and processed products. In the PCA score plot, samples were clustered into two major groups: S in one group, while St, L, and the raw MO in the other group. The PCA results were the same as those of HCA. The PCA loading scatters plot showed the correlation between the variables in the PC1 and PC2 coordinate systems and the association between the variables and the samples. From the PCA loading scatter plot, the classification was influenced by all nine GFns components (Figure S4). Among the nine GFns components, the variation trends of GF1 and GF2, GF7 and GF8 were consistent, while the variation trends of GF1 and GF2 were opposite to those of GF7 and GF8. Next, OPLS-DA was performed, which could find the greatest contributing constituents to the differences between the raw and processed products. As shown in Figure 6, these four groups (raw, St, S, and L) clustered separately in the OPLS-DA score plot, indicating a significant chemical variation between the raw and processed products. In the OPLS-DA model, the parameters *R*^2^ and Q^2^ indicated the explanatory and predictive ability of the model, respectively. Both the two parameters were above 0.5 in this model (*R*^2^ = 0.74 and *Q*^2^ = 0.56, respectively), suggesting that the explanatory and predictive capacity of the established model was satisfactory. The permutation test was used to ascertain whether the model was over-fitted, and the intercept of *Q*^2^ on the *y*-axis in this model was less than 0.05, meaning that the model was not over-fitted.

**Figure 7:**
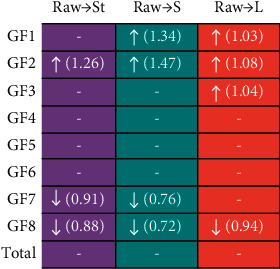
Overview graph of the ratio of the content for the processed products to the raw (the arrows indicate the change in content (increase or decrease) after processing, and the values in parentheses indicate the multiplicity of change).

The VIP values in the OPLS-DA model indicated the influence intensity and explanatory power of each metabolite effect on the sample classification, which could be used for dissecting the potential markers. In this work, the components with VIP values >1.0 were selected as differential compounds between the raw and processed products. The results showed that the magnitude of VIP values of differential compounds for raw-St, raw-S, and raw-L were GF2 > GF8 > GF7, GF2 > GF1 > GF8 > GF7, and GF2 > GF8 > GF1 > GF3, respectively (Figure S5). Subsequently, we further analyzed the content ratio of GF1, GF2, GF3, GF7, and GF8 for the processed products to the raw (Figure 7). Compared to the raw MO, the content of GF8 in L decreased slightly, and the content of GF1, GF2, and GF3 increased slightly. Nevertheless, compared to the raw, the contents of GF7 and GF8 in S and St were significantly decreased, and the contents of GF2 were significantly increased. The difference in the content of oligosaccharides between the processed products and the raw may come from the different processing methods, where St and S were steaming while L was boiling. There were two possible reasons for the insignificant changes of GFns content in L: (1) GFns with a high degree of polymerization may be hydrolyzed at high temperatures. The higher the temperature was, the stronger the hydrolysis was. In the steaming-process (S and St), when the temperature was greater than 100°C, the hydrolysis reaction of GFns was strong and the content changed greatly. However, in the boiling-process (L), when the temperature was about 100°C, the hydrolysis reaction of GFns was weak. (2) The procedure of the preparation of L consisted of two steps, first, decocting licorice and removing the residue for the decoction, and then boiling the raw MO with the decoction. The increase of the GF3 content in L may be due to the increase in its solubility caused by the addition of licorice [27]. Although both St and S were obtained by steaming-process, the change in the content of S was more significant than that of St, which may be due to the addition of salt.

## 4. Conclusions

In this study, the calculation methods of RCFs in the HILIC-HPLC-ELSD system were first investigated by six methods, using eight GFns in MO as an example. It was found that only one method was scientific. It was much important for developing the QAMS method for the Pharmacopoeia method when performing the LC-ELSD method. Based on the selected calculation method of RCF, seven fructooligosaccharides of *Morinda officinalis* were determined and validated with nystose (GF3) as a single standard. It was found that the contents of GF3 in the raw and processed MO were about 5%, while the total content of the eight oligosaccharides was about 45%, indicating that it was important to evaluate the quality using the eight GFns rather than GF3. Finally, the decrease of GF7 and GF8 and the increase of GF1 and GF2 during the steaming-process (S and St) and boiling-process(L) were revealed by the chemometrics.

It could be concluded that during the processing of MO, among the eight fructooligosaccharides, the content of GF7 and GF8 decreased while the content of GF1 and GF2 increased. It might be that during heat-processing, GF7 and GF8 with a high degree of polymerization degraded into GF1 and GF2 with a low degree of polymerization. The content variations of GFns before and after processing might be related to the processing method (steaming or boiling) and the addition of the excipients (salt or licorice). For L, the content variations of GFns before and after processing were less than those of S and St, which were steamed and salt-steamed. It might be that the heating temperature during boiling was slightly lower than that during steaming and the addition of licorice improved the solubility of GFns. For S and St, probably due to the addition of salt, the content variations of GFns in St before and after processing were slightly less than that in S.

## Figures and Tables

**Table 1:** The information of 30 batches of raw and processed MO samples.

No.	Batch number	Type	Origin
1	Raw_01	Raw	Guangdong
2	Raw_02	Raw	Guangdong
3	Raw_03	Raw	Guangxi
4	St_01-1	Steam-processed	Raw_01
5	St_01-2	Steam-processed	Raw_01
6	St_01-3	Steam-processed	Raw_01
7	St_02-1	Steam-processed	Raw_02
8	St_02-2	Steam-processed	Raw_02
9	St_02-3	Steam-processed	Raw_02
10	St_03-1	Steam-processed	Raw_03
11	St_03-2	Steam-processed	Raw_03
12	St_03-3	Steam-processed	Raw_03
13	L_01-1	Licorice-processed	Raw_01
14	L_01-2	Licorice-processed	Raw_01
15	L_01-3	Licorice-processed	Raw_01
16	L_02-1	Licorice-processed	Raw_02
17	L_02-2	Licorice-processed	Raw_02
18	L_02-3	Licorice-processed	Raw_02
19	L_03-1	Licorice-processed	Raw_03
20	L_03-2	Licorice-processed	Raw_03
21	L_03-3	Licorice-processed	Raw_03
22	S_01-1	Salt-processed	Raw_01
23	S_01-2	Salt-processed	Raw_01
24	S_01-3	Salt-processed	Raw_01
25	S_02-1	Salt-processed	Raw_02
26	S_02-2	Salt-processed	Raw_02
27	S_02-3	Salt-processed	Raw_02
28	S_03-1	Salt-processed	Raw_03
29	S_03-2	Salt-processed	Raw_03
30	S_03-3	Salt-processed	Raw_03

**Table 2:** The six methods for calculating the relative correction factor.

Method	Formula of RCF
A [22]	lgA_k_ = lgC_k−detected_ × F_s_ + *b*^*∗*^
	RCF^#^ = C_k−detected_/C_k_
B [23, 24]	RCF = *F*_k_/*F*_s_
C [25]	RCF^#^ = (*C*_s_/lgA_s_)/(*C*_k_/lgA_k_)
D [3]	RCF^#^ = (lgC_k_/lgA_k_)/(lgC_s_/lgA_s_)
E [26]	RCF^#^ = (A_s_/*C*_s_)/(A_k_/*C*_k_)
F [22]	RCF = 1.00

^*∗*^ *b* was the intercept of the linear equation for the single marker. ^#^The RCF of each analyte was the average of RCFs at different concentration levels.

**Table 3:** The results of calibration curves and linear range.

Components	Regression curve	*R * ^2^	Linear range (*μ*g/mL)	LOD (*μ*g/mL)	LOQ (*μ*g/mL)
GF1	*y* = 1.88*x* − 1.70	0.9998	116.77–934.13	1.30	4.32
GF2	*y* = 1.94*x* − 1.90	1	73.04–730.42	2.59	8.62
GF3	*y* = 1.82*x* − 1.67	0.9998	118.04–1180.40	1.86	6.21
GF4	*y* = 1.80*x* − 1.56	0.9999	95.04–950.40	1.98	6.59
GF5	*y* = 1.81*x* − 1.66	1	113.09–1130.92	2.08	6.92
GF6	*y* = 1.81*x* − 1.76	1	121.22–1212.15	2.47	8.23
GF7	*y* = 1.83*x* − 1.77	0.9999	116.24–1162.39	2.64	8.81
GF8	*y* = 1.86*x* − 1.91	0.9999	103.58–1035.75	3.41	11.37

**Table 4:** The RCFs of the eight compounds against GF3.

Method	GF1	GF2	GF3	GF4	GF5	GF6	GF7	GF8
A	0.86	0.91	1.00	0.92	1.04	1.16	1.11	1.18
B	1.03	1.07	1.00	0.99	0.99	1.00	1.00	1.02
C	1.17	1.44	1.00	1.20	1.02	0.94	0.98	1.05
D	0.97	1.03	1.00	1.00	1.01	1.04	1.03	1.06
E	0.87	1.28	1.00	1.03	1.10	1.27	1.23	1.51
F	1.00	1.00	1.00	1.00	1.00	1.00	1.00	1.00

**Table 5:** The results of precisions, stability, and repeatability.

No.	Components	Precision (RSD%) (*n* = 9)	Stability (0, 6, 12, 24, 36 h) (RSD%)		Repeatability (RSD%) (*n* = 9)
			Sample solution	Standard solution	
1	GF2	0.94	1.23	2.23	1.80
2	GF3	0.96	1.81	0.85	2.43
3	GF4	1.83	2.41	1.00	2.45
4	GF5	0.76	1.47	1.02	2.15
5	GF6	0.68	1.31	1.70	1.34
6	GF7	1.69	1.80	1.28	2.69
7	GF8	1.91	2.62	0.80	3.22

**Table 6:** The results of recovery for GF3.

Level	Amount (g)	Origin (mg)	Spike (mg)	Detected (mg)	Recovery (%)	RSD (%)
Low	0.1248	4.892	2.5	7.285	95.72	1.81
	0.1249	4.896	2.5	7.341	97.82	
	0.1249	4.896	2.5	7.377	99.24	

Medium	0.1253	4.912	5.0	9.966	101.09	1.81
	0.1255	4.920	5.0	10.12	104.10	
	0.1252	4.908	5.0	9.945	100.75	

High	0.1256	4.924	7.5	12.80	104.96	1.59
	0.1252	4.908	7.5	12.56	102.09	
	0.1255	4.920	7.5	12.79	104.96	

**Table 7:** The mean RCFs and their RSDs of eight GFns detected by different instruments (*n* = 2).

No.	Components	HPLC system	
		RCFs	RSD (%)
1	GF1	0.86	0.2
2	GF2	0.91	0.5
3	GF3	1.00	0.0
4	GF4	0.93	1.2
5	GF5	1.05	1.4
6	GF6	1.15	0.1
7	GF7	1.12	1.3
8	GF8	1.18	1.8

## Data Availability

The data used to support the findings of this study are available from the corresponding authors upon request.

## Supplementary Materials

Supplementary Materials Table S1. Comparison of the content determined by QAMS (Method A) and ESM in raw and processed products (%, g/g) (*n* = 2). Figure S1. *Morinda officinalis* Radix (MO, Raw) and its processing procedures: Steam-processed MO (StMO, St), Salt-processed MO (SMO, S), and Licorice-processed MO (LMO, L). Figure S2. The chromatographic separation of GFns on three columns. (A)-ACHROM XAmide (4.6 × 150 mm, 5 *μ*m (100A)), (B)-ZIC HILIC (4.6 × 250 mm, 5 *μ*m (200A)), and (C)-XBridge HILIC (4.6 × 250 mm, 5 *μ*m). Figure S3. Ten batches of Raw (02) and its processed products, two of which had abnormal values, *i*. e. S_02-3 and L_02-3. Figure S4. HCA results (A), PCA score plots (B), and loadings scatter plots (C) for Raw, St, S, and L. Figure S5. OPLS-DA score plots of Raw, St, S, and L.
