# Supplementary material for: Calculating Relative Correction Factors for Quantitative Analysis with HILIC-HPLC-ELSD Method: Eight Fructooligosaccharides of Morinda Officinalis as a Case Study
Source: J Anal Methods Chem. 2022 Aug 12;2022:8022473. doi: 10.1155/2022/8022473 (PMC9391178; doi:10.1155/2022/8022473)
Supplement: Supplementary Materials — Table S1. Comparison of the content determined by QAMS (Method A) and ESM in raw and processed products (%, g/g) (n = 2). Figure S1. Morinda officinalis Radix (MO, Raw) and its processing procedures: Steam-processed MO (StMO, St), Salt-processed MO (SMO, S), and Licorice-processed MO (LMO, L). Figure S2. The chromatographic separation of GFns on three columns. (A)-ACHROM XAmide (4.6 × 150 mm, 5 μm (100A)), (B)-ZIC HILIC (4.6 × 250 mm, 5 μm (200A)), and (C)-XBridge HILIC (4.6 × 250 mm, 5 μm). Figure S3. Ten batches of Raw (02) and its processed products, two of which had abnormal values, i. e. S_02-3 and L_02-3. Figure S4. HCA results (A), PCA score plots (B), and loadings scatter plots (C) for Raw, St, S, and L. Figure S5. OPLS-DA score plots of Raw, St, S, and L. [file 8022473.f1.docx]

# Journal of Analytical Methods in Chemistry

# Calculating relative correction factors for quantitative analysis with HILIC-HPLC-ELSD method: Eight Fructooligosaccharides of *Morinda officinalis* as a case study

Lihong Zhou,^1,3 #^ Hui Ni,^2,3 #^ Linlin Zhang,^3 #^ Wenyong Wu,^2,3^ Tengqian Zhang,^3,4^ Qi Su,^2,3^ Jing Zhou,^3^ Huali Long,^3^ Jinjun Hou,^3^ Jiyu Gong,^1^ and Wanying Wu^3^

^1^ College of Pharmacy, Changchun University of Chinese Medicine, Changchun 130117, China.
^2^ School of Chinese Materia Medica, Nanjing University of Chinese Medicine, Nanjing 210029, China. ^3^ National Engineering Research Center of TCM Standardization Technology, Shanghai Institute of Materia Medica, Chinese Academy of Sciences, Shanghai 201203, China.
^4^ University of Chinese Academy of Sciences, Beijing 100049, China.

^#^ Both the authors contributed equally to this work.

*** Corresponding author**

Wanying Wu,

E-mail address: wanyingwu@simm.ac.cn

Mailing address: 501 Haike Road, Shanghai 201203, China

Tel: +86-21- 50272789; Fax: +86-21- 50272789

National Engineering Research Center of TCM Standardization Technology, Shanghai Institute of Materia Medica, Chinese Academy of Sciences, Shanghai 201203, China.

Jiyu Gong,

E-mail address: [gjy0431@126.com](mailto:gjy0431@126.com).

Mailing address: 1035 Boshuo Road, Changchun 130117, China

College of Pharmacy, Changchun University of Chinese Medicine, Changchun, P.R. China.

## Supplementary Materials

Table S1. Comparison of the content determined by QAMS (Method A) and ESM in raw and processed products (%, g/g) (n=2).

Figure S1. *Morinda officinalis* Radix (MO, Raw) and its processing procedures: Steam-processed MO (StMO, St), Salt-processed MO (SMO, S), and Licorice-processed MO (LMO, L).

Figure S2. The chromatographic separation of GFns on three columns. (A)-ACHROM XAmide (4.6*150 mm, 5 μm (100A)), (B)-ZIC HILIC (4.6*250 mm, 5 μm (200A)), and (C)-XBridge HILIC (4.6*250 mm, 5 μm)

Figure S3. Ten batches of Raw (02) and its processed products, two of which had abnormal values, i. e. S_02-3 and L_02-3.

Figure S4. HCA results (A), PCA score plots (B), and loadings scatter plots (C) for Raw, St, S, and L.

Figure S5. OPLS-DA score plots of Raw, St, S, and L.

Table S1. Comparison of the content determined by QAMS (Method A) and ESM in raw and processed products (%, g/g) (n=2)..

| Sample no. | **GF1** | | RE% | **GF2** | | RE% | **GF3** | | RE% | **GF4** | | RE% | **GF5** | | RE% | **GF6** | | RE% | **GF7** | | RE% | **GF8** | | RE% | **Total** | | RE% |
| --- | --- | --- | --- | --- | --- | --- | --- | --- | --- | --- | --- | --- | --- | --- | --- | --- | --- | --- | --- | --- | --- | --- | --- | --- | --- | --- | --- |
|  | ESM | QAMS |  | ESM | QAMS |  | ESM | QAMS |  | ESM | QAMS |  | ESM | QAMS |  | ESM | QAMS |  | ESM | QAMS |  | ESM | QAMS |  | ESM | QAMS |  |
| Raw_01 | 2.99 | 2.96 | -1.04 | 1.69 | 1.60 | -5.34 | 4.25 | 4.25 | - | 5.27 | 5.26 | -0.28 | 6.48 | 6.46 | -0.24 | 7.21 | 7.20 | -0.16 | 6.22 | 6.23 | 0.16 | 5.73 | 5.69 | -0.62 | 39.85 | 39.66 | -0.47 |
| Raw_02 | 4.32 | 4.33 | 0.05 | 2.49 | 2.42 | -3.02 | 5.91 | 5.91 | - | 7.12 | 7.08 | -0.52 | 8.43 | 8.39 | -0.39 | 8.74 | 8.72 | -0.22 | 7.13 | 7.15 | 0.21 | 6.16 | 6.13 | -0.51 | 50.31 | 50.14 | -0.36 |
| Raw_03 | 3.25 | 3.22 | -0.86 | 1.88 | 1.79 | -4.85 | 5.69 | 5.69 | - | 7.11 | 7.08 | -0.52 | 8.60 | 8.57 | -0.41 | 9.26 | 9.23 | -0.25 | 7.87 | 7.89 | 0.26 | 7.00 | 6.99 | -0.20 | 50.66 | 50.45 | -0.41 |
| St_01-1 | 3.44 | 3.42 | -0.62 | 2.12 | 2.04 | -3.94 | 4.66 | 4.66 | - | 5.55 | 5.54 | -0.32 | 6.64 | 6.62 | -0.25 | 7.21 | 7.20 | -0.15 | 6.13 | 6.14 | 0.15 | 5.51 | 5.47 | -0.72 | 41.26 | 41.08 | -0.44 |
| St_01-2 | 3.25 | 3.22 | -0.78 | 2.14 | 2.06 | -3.84 | 4.42 | 4.42 | - | 5.26 | 5.25 | -0.28 | 6.37 | 6.36 | -0.22 | 6.89 | 6.88 | -0.14 | 5.88 | 5.89 | 0.13 | 5.31 | 5.27 | -0.80 | 39.52 | 39.33 | -0.46 |
| St_01-3 | 3.18 | 3.15 | -0.85 | 2.03 | 1.95 | -4.19 | 4.49 | 4.49 | - | 5.42 | 5.40 | -0.31 | 6.52 | 6.51 | -0.24 | 7.05 | 7.04 | -0.15 | 6.02 | 6.03 | 0.15 | 5.40 | 5.36 | -0.76 | 40.12 | 39.93 | -0.47 |
| St_02-1 | 4.57 | 4.59 | 0.33 | 3.24 | 3.21 | -1.08 | 6.49 | 6.49 | - | 7.33 | 7.28 | -0.57 | 8.35 | 8.32 | -0.41 | 8.15 | 8.13 | -0.21 | 6.35 | 6.36 | 0.17 | 5.27 | 5.23 | -0.80 | 49.75 | 49.61 | -0.29 |
| St_02-2 | 4.45 | 4.46 | 0.26 | 3.24 | 3.21 | -1.06 | 6.47 | 6.47 | - | 7.29 | 7.25 | -0.57 | 8.33 | 8.30 | -0.41 | 8.15 | 8.14 | -0.21 | 6.35 | 6.36 | 0.17 | 5.29 | 5.25 | -0.78 | 49.57 | 49.43 | -0.29 |
| St_02-3 | 4.58 | 4.60 | 0.35 | 3.36 | 3.34 | -0.83 | 6.47 | 6.47 | - | 7.25 | 7.21 | -0.57 | 8.31 | 8.27 | -0.41 | 8.15 | 8.13 | -0.21 | 6.32 | 6.33 | 0.17 | 5.31 | 5.27 | -0.78 | 49.75 | 49.62 | -0.27 |
| St_03-1 | 3.16 | 3.13 | -0.87 | 2.28 | 2.20 | -3.45 | 5.61 | 5.61 | - | 6.70 | 6.66 | -0.49 | 7.86 | 7.83 | -0.36 | 8.24 | 8.22 | -0.21 | 6.73 | 6.74 | 0.20 | 5.83 | 5.80 | -0.58 | 46.40 | 46.19 | -0.44 |
| St_03-2 | 3.15 | 3.12 | -0.86 | 2.28 | 2.20 | -3.40 | 5.72 | 5.72 | - | 6.84 | 6.81 | -0.52 | 8.09 | 8.06 | -0.39 | 8.46 | 8.44 | -0.22 | 6.96 | 6.97 | 0.21 | 6.04 | 6.01 | -0.48 | 47.53 | 47.32 | -0.43 |
| St_03-3 | 2.98 | 2.95 | -1.06 | 2.30 | 2.22 | -3.39 | 5.67 | 5.67 | - | 6.77 | 6.74 | -0.50 | 8.00 | 7.98 | -0.37 | 8.29 | 8.27 | -0.21 | 6.82 | 6.83 | 0.20 | 5.89 | 5.86 | -0.56 | 46.73 | 46.52 | -0.45 |
| L_01-1 | 4.53 | 4.54 | 0.23 | 2.74 | 2.68 | -2.34 | 5.09 | 5.09 | - | 5.44 | 5.42 | -0.29 | 6.10 | 6.09 | -0.18 | 6.07 | 6.06 | -0.08 | 4.82 | 4.82 | 0.04 | 4.08 | 4.02 | -1.45 | 38.87 | 38.73 | -0.37 |
| L_01-2 | 5.18 | 5.21 | 0.67 | 2.94 | 2.89 | -1.86 | 4.86 | 4.86 | - | 4.93 | 4.92 | -0.21 | 5.36 | 5.35 | -0.10 | 5.25 | 5.25 | -0.02 | 4.16 | 4.16 | -0.03 | 3.54 | 3.47 | -1.78 | 36.21 | 36.11 | -0.28 |
| L_01-3 | 4.60 | 4.61 | 0.29 | 2.59 | 2.52 | -2.70 | 4.68 | 4.68 | - | 4.96 | 4.95 | -0.21 | 5.52 | 5.51 | -0.12 | 5.60 | 5.60 | -0.05 | 4.48 | 4.48 | 0.00 | 3.81 | 3.75 | -1.60 | 36.25 | 36.11 | -0.38 |
| L_02-1 | 5.47 | 5.52 | 0.86 | 3.55 | 3.53 | -0.59 | 6.55 | 6.55 | - | 6.86 | 6.82 | -0.50 | 7.59 | 7.57 | -0.33 | 7.16 | 7.15 | -0.15 | 5.39 | 5.40 | 0.09 | 4.34 | 4.29 | -1.29 | 46.92 | 46.83 | -0.20 |
| L_02-2 | 4.21 | 4.21 | -0.04 | 2.88 | 2.82 | -2.09 | 6.01 | 6.01 | - | 6.62 | 6.59 | -0.46 | 7.51 | 7.49 | -0.32 | 7.54 | 7.53 | -0.16 | 6.04 | 6.05 | 0.14 | 5.19 | 5.14 | -0.91 | 45.99 | 45.83 | -0.36 |
| L_02-3 | 2.95 | 2.92 | -1.12 | 1.73 | 1.64 | -5.30 | 5.28 | 5.28 | - | 6.64 | 6.61 | -0.47 | 8.10 | 8.07 | -0.38 | 8.71 | 8.69 | -0.23 | 7.34 | 7.36 | 0.23 | 6.50 | 6.48 | -0.35 | 47.26 | 47.05 | -0.45 |
| L_03-1 | 4.15 | 4.14 | -0.07 | 2.74 | 2.68 | -2.37 | 5.71 | 5.71 | - | 6.28 | 6.25 | -0.42 | 7.12 | 7.10 | -0.28 | 7.14 | 7.13 | -0.14 | 5.70 | 5.70 | 0.11 | 4.80 | 4.75 | -1.08 | 43.62 | 43.45 | -0.39 |
| L_03-2 | 4.43 | 4.44 | 0.15 | 2.87 | 2.81 | -2.06 | 5.92 | 5.92 | - | 6.52 | 6.49 | -0.45 | 7.34 | 7.31 | -0.30 | 7.47 | 7.46 | -0.16 | 6.00 | 6.01 | 0.13 | 5.08 | 5.03 | -0.95 | 45.63 | 45.48 | -0.34 |
| L_03-3 | 4.27 | 4.27 | 0.02 | 2.84 | 2.78 | -2.14 | 6.13 | 6.13 | - | 6.84 | 6.81 | -0.49 | 7.83 | 7.80 | -0.35 | 7.94 | 7.93 | -0.19 | 6.42 | 6.43 | 0.16 | 5.45 | 5.41 | -0.79 | 47.72 | 47.56 | -0.35 |
| S_01-1 | 3.72 | 3.71 | -0.43 | 1.88 | 1.79 | -4.86 | 4.61 | 4.61 | - | 5.59 | 5.58 | -0.31 | 6.77 | 6.75 | -0.25 | 7.42 | 7.41 | -0.16 | 6.30 | 6.31 | 0.15 | 5.57 | 5.52 | -0.75 | 41.85 | 41.66 | -0.44 |
| S_01-2 | 3.04 | 3.00 | -1.11 | 1.73 | 1.63 | -5.46 | 4.14 | 4.14 | - | 5.16 | 5.15 | -0.23 | 6.42 | 6.41 | -0.20 | 7.03 | 7.02 | -0.13 | 5.94 | 5.95 | 0.12 | 5.27 | 5.22 | -0.90 | 38.73 | 38.53 | -0.52 |
| S_01-3 | 3.54 | 3.51 | -0.61 | 1.90 | 1.81 | -4.81 | 4.89 | 4.89 | - | 5.97 | 5.95 | -0.36 | 7.20 | 7.18 | -0.29 | 7.82 | 7.81 | -0.18 | 6.58 | 6.60 | 0.17 | 5.84 | 5.80 | -0.64 | 43.75 | 43.55 | -0.45 |
| S_02-1 | 4.04 | 4.03 | -0.15 | 2.83 | 2.77 | -2.17 | 6.15 | 6.15 | - | 7.13 | 7.09 | -0.53 | 8.41 | 8.38 | -0.40 | 8.56 | 8.54 | -0.22 | 6.86 | 6.88 | 0.20 | 5.87 | 5.84 | -0.61 | 49.86 | 49.68 | -0.36 |
| S_02-2 | 4.88 | 4.90 | 0.42 | 2.75 | 2.69 | -2.42 | 5.91 | 5.91 | - | 6.76 | 6.73 | -0.47 | 7.87 | 7.84 | -0.34 | 7.83 | 7.81 | -0.18 | 6.16 | 6.17 | 0.14 | 5.17 | 5.12 | -0.94 | 47.33 | 47.17 | -0.33 |
| S_02-3 | 2.02 | 1.97 | -2.38 | 1.36 | 1.27 | -6.91 | 3.82 | 3.82 | - | 4.97 | 4.96 | -0.21 | 6.23 | 6.22 | -0.19 | 6.77 | 6.77 | -0.12 | 5.78 | 5.79 | 0.11 | 5.26 | 5.22 | -0.88 | 36.22 | 36.01 | -0.58 |
| S_03-1 | 3.06 | 3.03 | -1.04 | 1.96 | 1.88 | -4.55 | 5.80 | 5.80 | - | 7.20 | 7.16 | -0.53 | 8.65 | 8.61 | -0.41 | 9.18 | 9.16 | -0.24 | 7.57 | 7.58 | 0.24 | 6.64 | 6.62 | -0.33 | 50.06 | 49.84 | -0.44 |
| S_03-2 | 3.13 | 3.10 | -0.97 | 2.02 | 1.93 | -4.38 | 5.98 | 5.98 | - | 7.31 | 7.27 | -0.55 | 8.88 | 8.84 | -0.43 | 9.45 | 9.43 | -0.25 | 7.86 | 7.88 | 0.26 | 6.90 | 6.88 | -0.24 | 51.52 | 51.30 | -0.42 |
| S_03-3 | 2.80 | 2.76 | -1.34 | 1.86 | 1.76 | -4.94 | 5.62 | 5.62 | - | 6.98 | 6.94 | -0.50 | 8.52 | 8.48 | -0.40 | 9.12 | 9.09 | -0.24 | 7.63 | 7.65 | 0.24 | 6.79 | 6.77 | -0.29 | 49.31 | 49.08 | -0.45 |


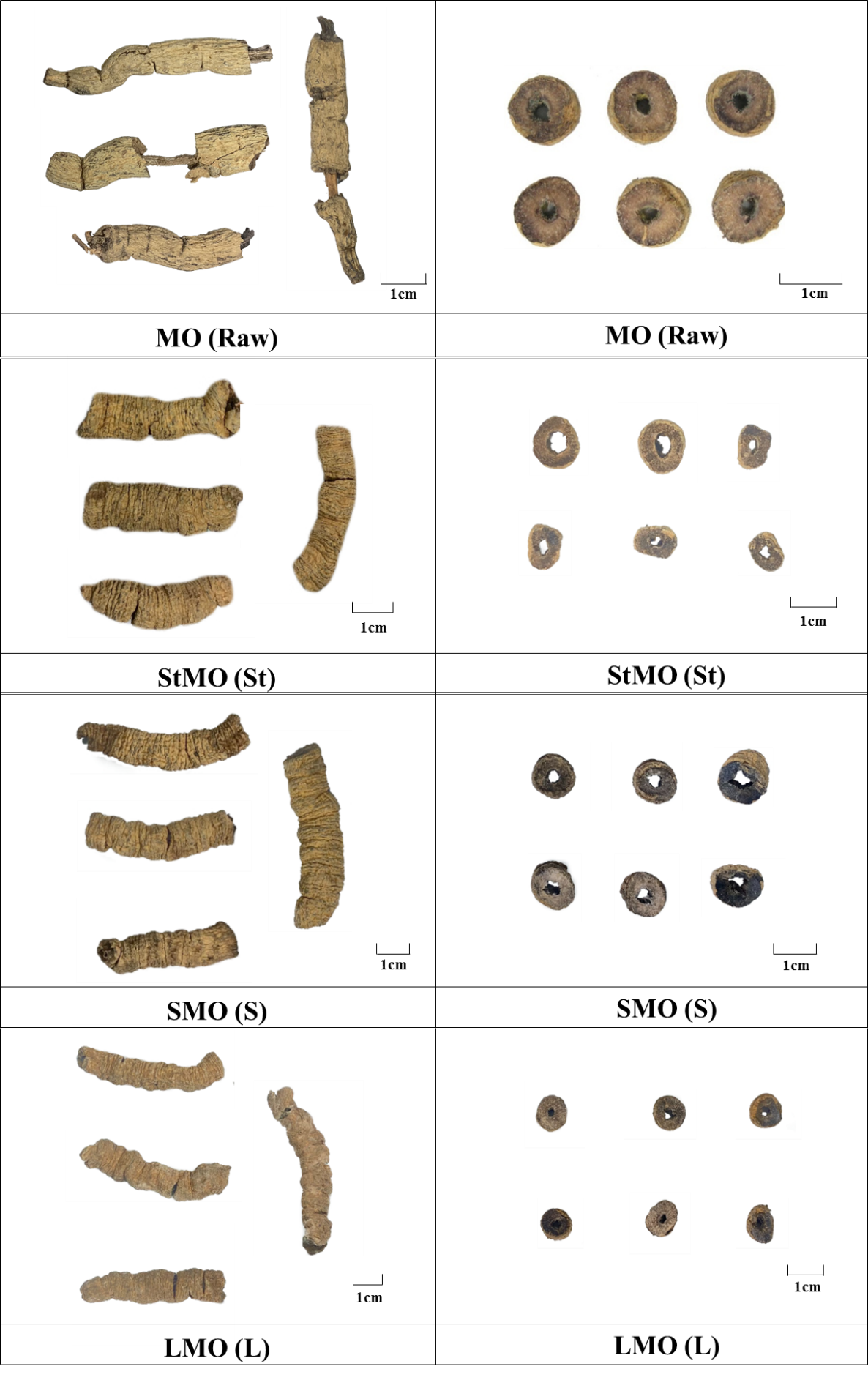


Figure S1. *Morinda officinalis Radix* (MO, Raw) and its processing procedures: Steam-processed MO (StMO, St), Salt-processed MO (SMO, S), and Licorice-processed MO (LMO, L).


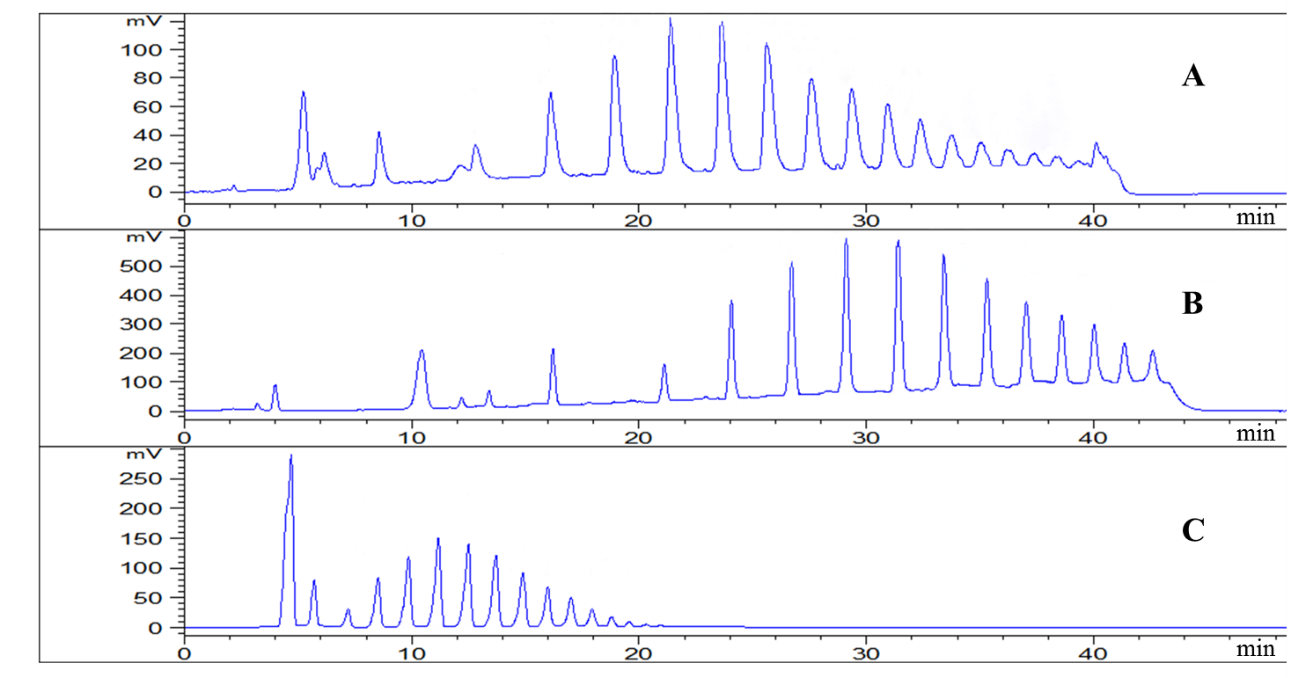


Figure S2. The chromatographic separation of GFns on three columns. (A)-ACHROM XAmide (4.6*150 mm, 5 μm (100A)), (B)-ZIC HILIC (4.6*250 mm, 5 μm (200A)), and (C)-XBridge HILIC (4.6*250 mm, 5 μm).


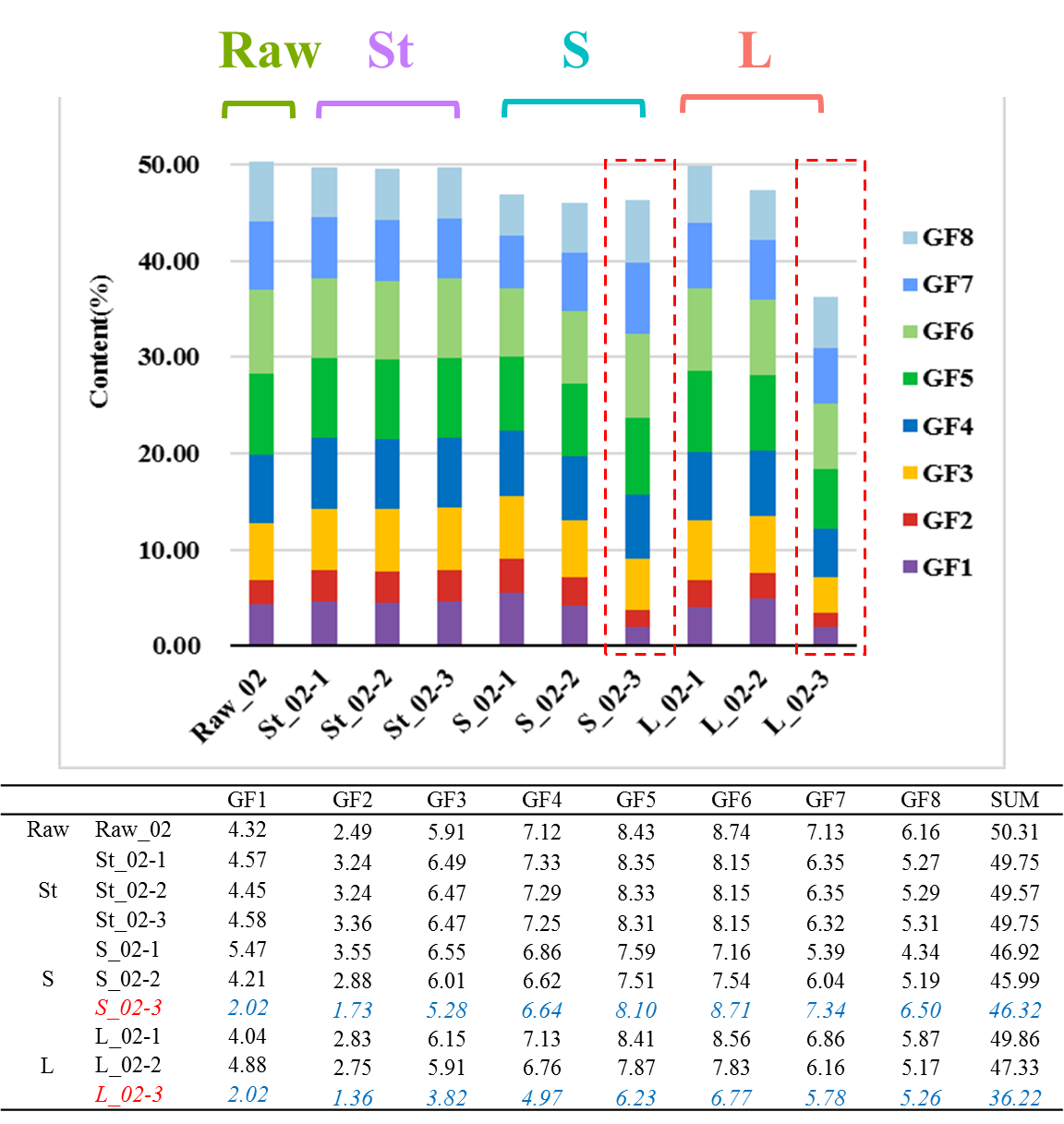


Figure S3. Ten batches of Raw (02) and its processed products, two of which had abnormal values, i. e. S_02-3 and L_02-3.


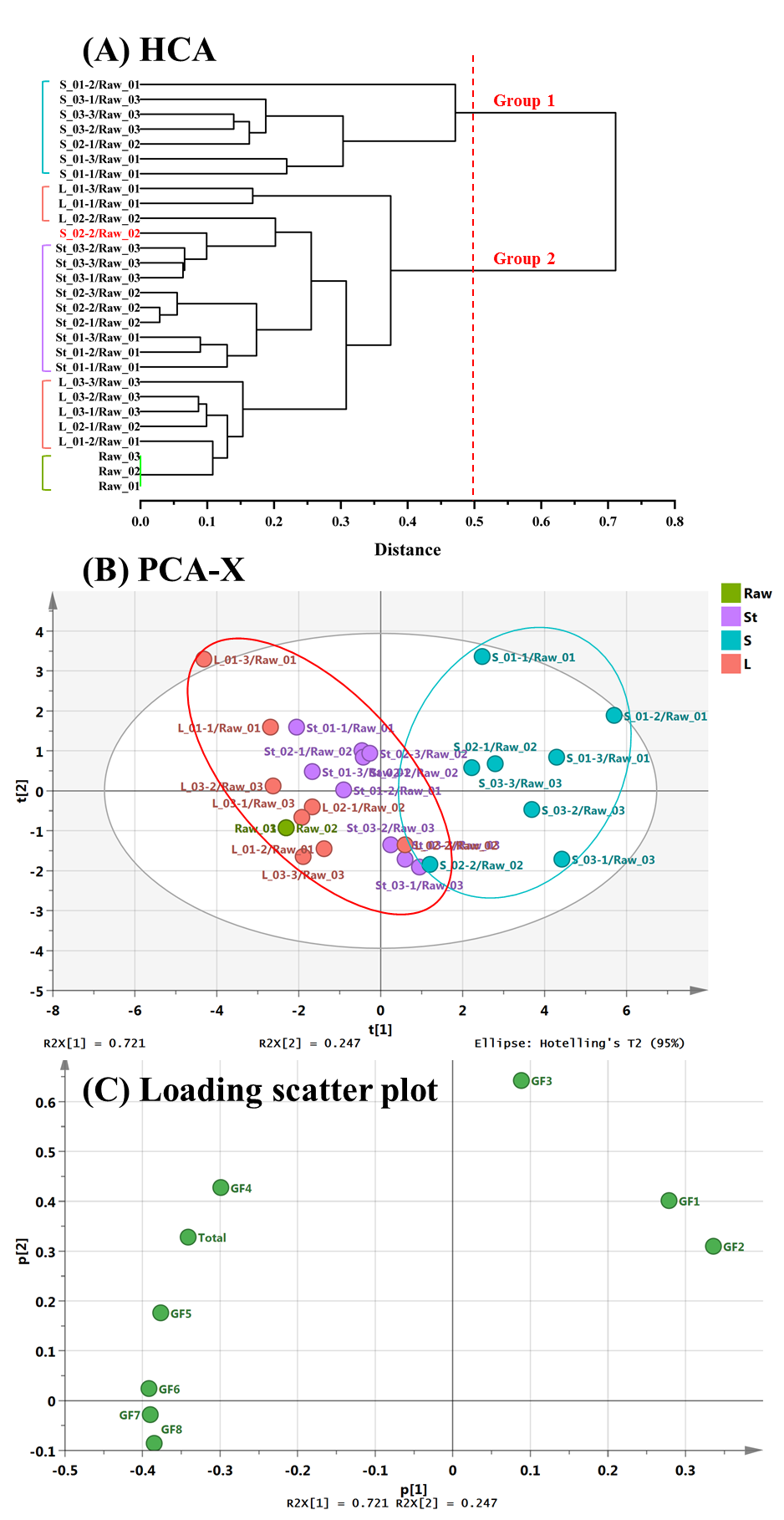


Figure S4. HCA results (A), PCA score plots (B), and loadings scatter plots (C) for Raw, St, S, and L.


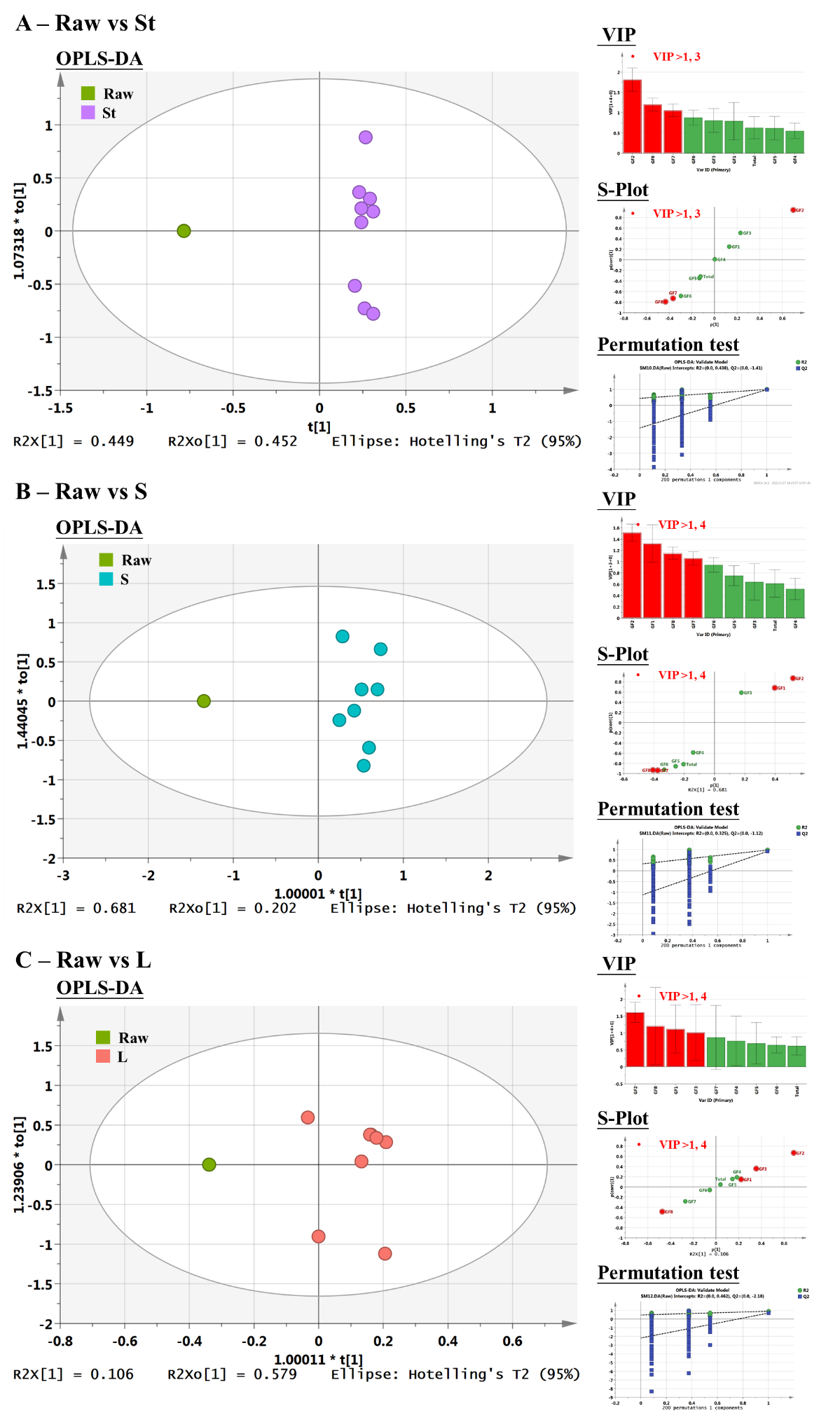


Figure S5. OPLS-DA score plots of Raw, St, S, and L.
